# Foreign Correspondence

**Published:** 1889-11

**Authors:** 


### Foreign Correspondence.

To the Editor:

The following case in practice may be of interest to others besides myself, because, there being three methods, by either one of which the articulation could have been restored, it presented the difficulty of deciding which method would, under the circumstances, be the best to adopt.

The patient is a German, and came to me about eighteen months ago, in order to consult with me as to the best means I would suggest for the regulation of his teeth. I had reason to believe he had consulted with others, and it was upon my decision that he consented to let me operate.

He had worn his teeth away in a manner that I have seldom seen. The chief sufferers being the lower molars, canines, and incisors, and the upper canines and bicuspids, disclosing, when his mouth was opened, a most irregular state of affairs. What really brought him to seek relief was the fact that the upper centrals were literally wounding the lower gums, so much so that he could not eat; otherwise, terribly worn as were his teeth, they did not pain him in the least. My first impulse was to effect a radical cure, and extract the majority, if not all, of the teeth, and make him an artificial denture complete. But a good-Samaritan kind of feeling came over me, and I thought, under the same circumstances, I should not like any one to suggest a similar thing to me in cold blood, if there was any other way of operating. Then I bethought me to kill the pulps of the worst teeth, and build them all up with gold tips; but what an operation for the poor patient,—what hours he must have spent with his mouth wide open, thinking as kindly (?) of me as he possibly could! At last it occurred to me to cap the teeth as they stood with gold, by which means I need not destroy a pulp, and, moreover, the greater part of the work could be done in the laboratory. I stated the plan to him, and he consented.

I first made the caps for the lower molars, opening the bite to its normal length, and when temporarily placed, I utilized the same sitting to make the caps for the lower canines; taking them off, they were finished ready for the next day. When he again presented himself, I fixed temporarily the four caps, and made two more,—one on either side,—for the lower bicuspids. In such manner all the caps were made to articulate, retaining the articulation in its natural position.

Then came a grand day, when, after making undercuts where I could with safety, I cemented the twelve caps or crowns into their positions at the same sitting, and extracted the roots of the incisors, they being so bad as to be useless for crowning. When the gums had thoroughly healed, I took an impression, from which I made him an artificial piece, in gold and vulcanite, to supply the place of the lost incisors. I think the method I employed—namely, of making all the caps first and cementing them on at one sitting—was the right practice; otherwise—and it would have occurred if I had decided to tip with gold—he would have passed some enjoyable (?) days first with one cap, then two, etc., entirely preventing him from eating until the whole of the work was complete.

I have seen him frequently since, and he says he eats with perfect ease with his golden teeth, and is thoroughly contented.

H. H. Edwards.

Madrid, Spain.

---

To the Editor:

The new officers of the American Dental Society of Europe are: President, Dr. Patton, of Cologne; Vice-President, Dr. Davenport, of Paris; Treasurer, Dr. Adams, of Frankfort on the Main; Secretary, Dr. Bryan, of Basel.

Owing to the International Medical Congress in Berlin, next August, which most of the members will attend, the next meeting is arranged for the first Tuesday in August, 1891, at Heidelberg. Personally I regret this delay, but those most interested in the Society voted in favor of it, and instanced the success of this year as evidence that a biennial meeting is not disadvantageous.

L. C. Bryan, *Secretary.*

Basel, Switzerland.

